# 

**Published:** 1877-11

**Authors:** 


### EXAMINERS' MEETING.

The Annual meeting of the Ohio State Board of Dental Examiners, will take place in Columbus on Tuesday the 4th of December, at 12 o'clock, M.

All those interested, and desiring to appear before the Board, should be promptly in attendance at the time indicated above; let there be no delay.

### OHIO DENTAL SOCIETY.

The Annual Meeting of the Ohio State Dental Society will be held in Columbus, on Wednesday, December 5th., when we hope to meet all the members of the Society, and all the members of the profession in the State, who feel an interest in our profession, and even if some have no higher motive than self interest, they will find it to their interest to be present.

The following subjects have been presented by the Executive Committed for essays and discussion, viz:

First. Influence of diseased teeth on other and remote organs.

Second. Sanitary treatment of teeth: therapeutic agents especially applicable in dental practice, their properties and uses.

Third. Lancing the gums during first dentition; is the operation called for? if so, how should it be performed?

Fourth. Mechanical dentistry.

Fifth. Modes of manipulating gold in filling teeth.

Sixth. Abrasion of the teeth; sensitiveness of the necks, causes and treatment.

It is desirable that there should be well written, comprehensive papers upon each of these subjects and preparation for good discussion. Also let every one come prepared to add something of interest and profit to the meeting, either in the way of new principles, new modes of practice, or improvements of old ones; or by the presentation of new or improved instruments and appliances.

We trust that the profession of the State will come out to this meeting in such numbers as to make it the largest one of this body ever held, and with such earnestness as to make it pre-eminently profitable.

A MONTHLY MAGAZINE DEVOTED TO THE INTERESTS OF

**THE DENTAL PROFESSION.**

*TERMS REDUCED TO $2.50 PER YEAR. SINGLE COPIES 25C.*

EDITED BY PROF. J. TAFT, D.D.S.

AND

*Published by SPENCER, CROCKER & CO., Ohio Dental Depot.*

The volume commences in January and closes in December.

The REGISTER being issued monthly is always fresh, therefore Indispensable to the practitioner who desires to keep posted up with the new inventions and improvements which are daily developed. Neither expense nor effort will be spared to give value and variety to its contents. Articles will be illustrated with well executed engravings whenever they will add to the interest or assist to explanation.

Its extensive circulation and frequency of issue make it one of the BEST DENTAL ADVERTISING MEDIUMS in the United States.

All subscriptions must begin with January or July.

Local or special notices, five lines or less, will be inserted for $3 each insertion; over five lines, 50 cts. per line.

All advertising bills payable in advance, or at furthest, after the issue of the first number containing the advertisement. All transient advertisements to be paid for in advance.

☞CONTRIBUTIONS SOLICITED.

Exclusively Editorial Communications should be addressed to Editor.

The latest number of the REGISTER may be ordered with or without other goods at any time, and all letters should be addressed to

*SPENCER, CROCKER & CO., Ohio Dental Depot, Cincinnati, O.*

